# A multilevel analysis of factors associated with unimmunized children in Ethiopia

**DOI:** 10.3389/fpubh.2025.1433606

**Published:** 2025-04-25

**Authors:** Getnet Mamo Habtie, Mequannent Sharew Melaku, Setognal Birara Aychiluhm

**Affiliations:** ^1^Department of Statistics, College of Natural and Computational Science, Samara University, Samara, Ethiopia; ^2^Department of Health Informatics, Institute of Public Health, University of Gondar, Gondar, Ethiopia; ^3^Department of Epidemiology and Biostatistics, Institute of Public Health, College of Medicine and Health Sciences, University of Gondar, Gondar, Ethiopia; ^4^Rural Health Research Institute, Charles Sturt University, Orange, NSW, Australia

**Keywords:** unimmunized children, multilevel, associated factors, Ethiopia, health survey data

## Abstract

**Introduction:**

In Ethiopia, 16% of deaths among children aged less than 5 years are attributable to diseases that can be prevented through vaccination. However, studies carried out in Ethiopia to date have not investigated how community- and individual-level factors affect children who receive no vaccine at all. Hence, this study aimed to assess the factors associated with unimmunized children in Ethiopia.

**Methods:**

This study is a secondary analysis of Demographic and Health Survey (DHS) data. A weighted sample of 6,219 children aged less than 5 years was included after a stratified two-stage cluster sampling. To determine the factors that influence unimmunized children, a multivariable multilevel logistic regression model was developed. In addition, adjusted odds ratio (AOR) with 95% confidence interval (CI) was used to select variables that have statistically significant effects on unimmunized children.

**Results:**

The prevalence of unimmunized children aged less than 5 years in Ethiopia was 22.45% (95% CI: 21.4–23.5). In the multivariable multilevel analysis, individual-level factors associated with increased odds of having unimmunized children included no media exposure (AOR 1.33, 95 CI: 1.02–1.63), place of delivery (AOR 1.5, CI: 1.22–1.91), and the child’s relationship to the household head—specifically between the household head and a daughter (AOR 1.5, 95 CI: 1.05–2.41) or categorized as others (AOR 1.8, 95, CI: 1.15–2.74). Community-level predictors associated with unimmunized children included place of residence (AOR 1.8, 95, CI: 1.12–2.58), distance from the health facility (AOR 1.32, 95, CI: 1.09–1.60), and community poverty level (AOR 1.4, 95, CI: 1.05–1.78).

**Conclusion:**

In Ethiopia, approximately one-fourth of children could not get basic vaccinations. Media exposure, place of delivery, relationship with the household head, place of residence, distance from the health facility, and community poverty were found to be factors significantly associated with unimmunized children.

## Background

One of the most effective public health initiatives for reducing childhood morbidity and mortality is immunization of children (1). It has been estimated that basic immunizations prevent 2.5 million child deaths globally each year (2, 3). The Expanded Programme on Immunization (EPI) was launched in 1974 to ensure that all children regularly obtain the recommended immunizations. However, many children below 5 years of age remain unvaccinated.

Immunization plays a vital role in improving public health worldwide, benefiting both developed and developing countries alike. It not only protects individuals from preventable diseases but also has significant societal and economic benefits. Thus, the implementation of effective immunization programs is crucial for reducing morbidity and mortality rates, promoting overall well-being, and advancing the development of these country (4, 5).

It is projected that, by 2055, the global population will approach approximately 10 billion, which is largely attributable to the effectiveness of vaccines in preventing diseases and thus extending life expectancy worldwide. However, substantial efforts are needed to guarantee the funding, delivery, distribution, and uptake of vaccines among all communities, especially among those that are remote, skeptical about vaccine efficacy, or affected by civil unrest (6).

In 1980, Ethiopia became one of the member states to adopt the EPI service as recommended by the WHO, with the aim of reducing child morbidity and mortality due to vaccine-preventable diseases (VPDs). However, the EPI faced challenges and did not achieve its intended success (7). Moreover, since 2003, Ethiopia has been following the Reaching Every District (RED) and Sustainable Outreach Services (SOS) approaches to improve immunization coverage (8).

Ethiopia provides the following basic: one dose of Bacillus Calmette–Guerin (BCG) and oral polio vaccine (OPV) given at birth; three doses each of each pentavalent, OPV, and pneumococcal conjugate vaccine (PCV) given at the 6th, 10th, and 14th weeks; and lastly measles vaccine at the 9th month (9–11).

Almost Approximately 29% of child fatalities can be prevented by vaccination (12). In 2011, one in out of five infants around the world did not receive life-saving vaccinations’ according to the statistics showing that 1.5 million children died and 22.4 million did not obtain the recommended three doses of DPT (13). Globally, 19.5 million children were incompletely immunized in 2016 (14). Approximately 60% of these children live in 10 countries, including Ethiopia (15). Furthermore, approximately one out of 10 infants lack access to any child vaccine worldwide (15).

In Ethiopia, infections that can be prevented by vaccination account for approximately 16% of deaths among children aged less than 5 years (16). The EPI service of the country aims to protect roughly 3 million newborns every year from VPDs (17), but a significant proportion of children remain unimmunized, which is the highest contributor to child mortality (mostly caused by VPDs) in Ethiopia (18, 19).

According to the 2016 Ethiopian Demographic and Health Survey (EDHS) data, full immunization coverage increased from 24% in 2011 to 39% in 2016 (19, 20); however, this achievement remains markedly below the goal set in sustainable development goals (11, 21). Even though EPI services are available to every kebele (i.e., a small administrative unit), children still remain unvaccinated in Ethiopia, with only 50% of children between 12 and 23 years of age receiving all of their recommended vaccinations (22).

Various studies have been carried out to determine the factors associated with immunization both in Ethiopia and worldwide (11, 23–33). However, these studies have seldom focused on how circumstances can affect children being unimmunized. In addition, using a single-level logistic regression analysis to examine data with a hierarchical structure (i.e., children nested within communities) violates the independence assumptions in basic logistic regression models (34, 35). Furthermore, even though there are a variety of interrelated characteristics associated with unvaccinated children, they are not always consistent throughout the study areas and researchers. Moreover, research prioritization is a dynamic and iterative process that requires ongoing review and updates as new findings are made and additional issues are identified, to further strengthen and document the already-existing evidence in support of the significant impact of individual- and contextual-level factors in the field of public health.

Therefore, to address the aforementioned challenges, we carried out this study to identify community- and individual-level factors associated with unimmunized children, which is a crucial first step in preventing child mortality.

## Methods

### Data and sample

This study is a secondary analysis of Demographic and Health Survey (DHS) data, which were sourced from the official database website of the DHS program.^1^ These data were collected during the period from 18 January 2016 to 27 June 2016. The EDHS is conducted every 5 years and is nationally representative. It covers Ethiopia’s nine regional states: (Afar; Amhara; Benishangul-Gumuz; Gambela; Harari; Oromia; Somali; Southern Nations, Nationalities, and People’s Region (SNNPR); and Tigray), as well as two administrative cities (Addis Ababa and Dire Dawa) (36).

The source population for this study comprised all children aged 0 to 59 months residing in Ethiopia, whereas the study population included all children less than 5 years of age residing in the chosen enumeration areas during EDHS data collection. A total of 6,219 children aged 0 to 59 months who met the inclusion criteria were included in this study. A two-stage probability sampling method, stratified by geographic region and urban/rural areas within each region, was used to select study participants, covering the entire target population of Ethiopia (37).

### Measures of variables

#### Dependent variable

Unimmunized children was the dependent variable with binary outcomes categorized into 1 = yes (children who had not received basic vaccination) and 0 = no (those who had received at least one dose of any basic vaccination and at most full dose of vaccination).

#### Independent variables

Independent variables included individual-level variables and community-level variables. The following are the individual-level variables: sociodemographic factors (mother’s age, mother’s educational level, marital status, sex of the household head, wealth index, occupation, religion, and relationship with the household head), obstetric factors (parity and place of delivery), and information communication (listening to the radio, watching television, reading the newspaper, and having a mobile phone). The following were the community-level variables: residence, region, distance from the health facility, community education, community poverty level, and community media exposure, among which the latter three variables were produced by aggregating individual-level variables.

### Model and data analysis

Based on the recommendation of the EDHS, proportions and frequencies were estimated after applying sample weights to the data to adjust for disproportionate sampling and non-responses since the allocation of the sample in the EDHS to different regions, as well as urban and rural areas, was non-proportional.

Variables were fitted into four models. To test for random variation in the intercept and calculate the intraclass correlation coefficient (ICC) and proportionate change in variance (PCV), model I (the empty model) was fitted without explanatory variables. Model II analyzed how individual-level traits affected individuals; model III analyzed how community-level factors affected individuals; and model IV (full model) analyzed how both individual- and community-level factors affected individuals simultaneously. The PCV and ICC were also used to measure variation. The Akaike Information Criterion (AIC) values were calculated for each model and compared, and the model with the lowest AIC value was chosen as the best-fit model.

As the full model (model with individual- and community-level variables) had the lowest AIC value, it was the model that best fit the data. AOR with 95% CI in the multivariable model was used to select variables that have a statistically significant association with unimmunized children.

## Results

### Sociodemographic characteristics of respondents

A total of 6,219 children aged less than 5 years were included in this study. Regarding maternal education, the majority of children (3,854, 61.97%) had no formal education, and the majority of children (5,500, 88.44%) were rural residents (Table 1). Regarding sex, 49.46% (3,076) were male and 50.54% (3,143) were female. The majority of mothers (51.75%, 3,218) were between 25 and 34 years of age, 27.43% (1,706) were between 15 and 24 years, and 20.82% (1,295) were between 35 and 49 years. In addition, 62.6% of mothers (3,894) were not employed, while 37.4% (2,324) were employed. In most of the cases, caregivers were the wives of the household heads (79.8%, 4,965). A small proportion of caregivers were daughters (5.5%, 338) or other relatives (4.8%, 299) such as daughter-in-law, granddaughter, and sister.

**Table 1:** Sociodemographic characteristics of respondents associated with unimmunized children in Ethiopia.

Variables	Frequency	Percentage
Sex of the child		
Male	3,076	49.46
Female	3,143	50.54
Age of the mother (in years)		
15–24	1706	27.43
25–34	3,218	51.75
35–49	1,295	20.82
Mother’s educational level		
No education	3,854	61.97
Primary education	1849	29.74
Secondary education/above	516	8.29
Sex of the household head		
Male	5,363	86.2
Female	854	13.8
Mother’s employment status		
Employed	2,324	37.4
Not employed	3,894	62.6
Residence		
Urban	719	11.56
Rural	5,500	88.44
Wealth status		
Poorest	1,504	24.2
Poorer	1,401	22.53
Middle	1,278	20.55
Richer	1,097	17.65
Richest	938	15.1
Father’s educational level		
No education	2,742	46.5
Primary	2,357	39.9
Secondary/above	801	13.6
Father’s employment status		
Employed	4,640	78.6
Not employed	1,260	21.4
Distance to the health facility		
Big challenge	3,764	60.52
Not big challenge	2,455	39.48
Has telephone		
Yes	100	1.6
No	619	98.4
Read magazine		
Yes	422	6.8
No	5,797	93.2
Listen to the radio		
Yes	1,693	27.2
No	4,526	72.8
Watch television		
Yes	1,141	13.34
No	5,078	81.66
Having a relationship with the household head		
Head	617	9.9
Wife	4,965	79.8
Daughter	338	5.5
Others	299	4.8

Others = daughter-in-law, granddaughter, sister, and other relatives.

In this study, 6,219 children and 6,217 caregivers were included. The sociodemographic characteristics of both children and caregivers were analyzed, highlighting the influence of key factors such as sex, age, educational level, and employment status on vaccination status. Children were nearly equally distributed between both sexes, while the majority of mothers were aged 25–34 years, with a significant proportion having no education. Households were predominantly rural, with the majority being male-headed households. Disparities were observed in wealth index and access to telecommunication tools, and healthcare access was limited due to the distance for many respondents (Table 1).

Several sociodemographic factors were associated with unimmunized children in Ethiopia. Children from rural areas, those from poorer households, and those whose mother had no formal education were more likely to be unimmunized. Maternal employment status influenced immunization rates, with children of unemployed mothers showing lower coverage. Household characteristics, such as male-headed households and perceived difficulty in accessing healthcare facilities, were also associated with lower immunization rates. In addition, limited media exposure (such as radio, television, or magazines) and lack of access to telecommunication devices further contributed to disparities in immunization coverage.

### Prevalence of unimmunized children

In Ethiopia, the prevalence of unimmunized children aged less than 5 years was 1,396 (22.45%) (95% CI: 21.4–23.5). A variation among unimmunized children was observed across regions of Ethiopia. The proportion of unimmunized children was lowest in Addis Ababa (3%) and highest in Afar (41%).

### Random effect model and model fitness

The community-level variance ICC was 46%, which indicates significant variation in unimmunized children across the enumeration areas. This difference declined from 46 to 23% when different variables were controlled; however, there were some variables that should be incorporated into the model to better explain the reset variability. In addition, the PCV result revealed that 61% of variation was explained by community-level variables, 60% by individual-level variables, and 71% by both individual-level and community-level variables. Based on the model comparisons, model IV was found to be the best-fit model, and therefore, interpretations were made for model IV (Table 2).

**Table 2:** Model comparison and model fitness results with respect to factors associated with unimmunized children in Ethiopia.

Measure of variation	Model I	Model II	Model III	Model IV
Random component	2.8	1.12	1.11	0.83
ICC	46	28	27	23
PCV	Ref	60	61	71
Model comparison				
AIC	5480.7	5,062	5,214	4965.5
Log likelihood	−2,738	−2505.05	−2590.95	−1671.7
DIC	5,476	5010.1	5,182	3343.5

### Factors associated with unimmunized children

The odds of having unimmunized children increased by 1.5 times (AOR 1.5, 95% CI 1.22–1.91) when women gave birth at home. It increased by 1.8 times (AOR 1.8, 95% CI 1.12–2.58) among rural women Compared to urban residents. In addition, the odds of having unimmunized children increased by 1.33 times (AOR 1.33, 95% CI 1.02–1.63) among women who had no media exposure compared to those who had exposure to media. Similarly, it increased by 1.4 times (AOR 1.4, 95% CI 1.05–1.78) among women from high-poverty communities compared to those from a low-poverty community. Among women who perceived distance from the health facility as a big challenge, the odds of having unimmunized children increased by 1.32 times (AOR 1.32, 95% CI 1.097–1.6) compared to those who did not. Furthermore, the odds of having unimmunized children among women who had low community media exposure increased by 1.9 times (AOR 1.9, 95% CI 1.39–2.58) compared to those who had high media exposure. Finally, the odds of having unimmunized children among women whose relationship with household head as a daughter increased by 1.5 times (AOR 1.5, 95% CI 1.05 2.41) and as others by 1.8 times (AOR 1.8, 95% CI 1.15–2.74) compared to women who were the household head (Table 3).

**Table 3:** Multilevel multivariable logistic regression of the individual- and community-level variables associated with unimmunized children in Ethiopia.

Variables	Model I	Model II	Model III	Model IV
Community media exposure				
Low			2.3 (1.8–3.4)	1.9 (1.39–2.58)*
High			1.00	1.00
Community education				
Low			1.46(1.03–2.08)*	1.1 (0.78–1.59)
High			1.00	1.00
Community poverty				
Low			1.00	1.00
High			1.96(1.38–2.79)*	1.4 (1.05–1.78)*
Distance from the health facility				
Big challenge			1.4(1.15–1.66)*	1.32(1.097–1.6)*
Not big challenge			1.00	1.00
Region				
Agrarian			1.4(0.86–2.33)	1.2(0.72–2.2)
Pastoralist			3(1.8–5)*	2.5(0.97- 3)
City			1.00	1.00
Residence				
Urban			1.00	1.00
Rural			2.2(1.34–3.52)*	1.8(1.12-2.58)*
Age of the mother				
15–24		1.3(0.996–1.645)		1.1 (0.88–1.43)
25–34		0.93(0.75–1.14)		0.9 (0.72–1.1)
35–49		1.00		1.00
Education level of the mother				
Not educated		1.74(1.2–2.66)*		1.4 (0.90–2.05)
Primary		1.2(0.7–1.7)		1.0 (0.67–1.46)
Secondary		1.00		1.00
Wealth index				
Poor		1.7(1.26–2.2)*		1.2 (0.94–1.63)
Middle		0.95(0.7–1.3)		0.8 (0.61–1.12)
Rich		1.00		1.00
Media exposure				1.00
No		1.55(1.114–2.2)*		1.3(1.02-1.63)*
Yes		1.00		1.00
Relationship with the household head				
Head		1.00		1.00
Wife		1(0.79–1.3)		1.1 (0.84–1.36)
Daughter		1.63(1.05–2.53)*		1.5 (1.05 2.41)*
Others		1.75(1.14-2.7)*		1.8 (1.15–2.74) **
Place of delivery				
Home		1.9(1.5–2.4)*		1.8(1.22-1.91)*
Health facility		1.00		1.00
Father’s education				
Not educated		0.99(0.74–1.34)		1.0 (0.74–1.33)
Primary		0.76(0.57–1.02)		0.8 (0.58–1.036)
Secondary and high		1.00		1.00
Has mobile phone				
No		1.76(0.75–4.15)		1.6 (0.66–3.87)
Yes		1.00		1.00
Religion				
Orthodox		1.00		1.00
Catholic		1.2(0.36–3.9)		0.6 (0.18–1.96)
Protestant		1.4(0.98–1.95)		0.8 (0.54–1.18)
Muslim		1.9(1.4–2.6)*		0.9 (0.65–1.35)
Others		2.2(1.1–4.1)*		1.3 (0.66–2.4)
Mother’s occupation				
Employed		1.00		1.00
Unemployed		1.2(0.98–1.43)		1.1 (0.92–1.35)

*Statistically significant variables at 95% confidence interval.

## Discussion

Unimmunized children remain a significant public health problem in Ethiopia even though efforts have been made to contain this issue. In this study, the prevalence of unimmunized children was found to be 22.45% (95% CI 21.4–23.5). In addition, the proportion of unimmunized children varied across the administrative regions of Ethiopia. The number of unimmunized children remains critically high and was not evenly distributed across the country. The EDHS reports also show only a slight reduction in the number of unimmunized children. A number of studies have also revealed that low immunization coverage across different parts of the country and in the entire country as well (23–25, 27). This high percentage of unimmunized children might be because Ethiopia is a developing country and its childhood vaccination strategies are poorly implemented by different agents working toward immunization services. In addition, inconsistent implementation of immunization services across the country might have led to a high percentage of unimmunized children. Furthermore, some areas are not accessible for transportation, which also contributes to the high percentage of unimmunized children.

After controlling for clustering/random effect, this study identified both individual- and community-level factors with a significant association with unimmunized children.

Among individual-level factors, media exposure, place of delivery, and relationship with the household head were significantly associated with unimmunized children. Among community-level factors, place of residence, distance from the health facility, media exposure, and community poverty were significantly associated with unimmunized children.

This study also revealed that home delivery had a significant positive association with unimmunized children. However, some studies have reported the negative impact of home delivery on vaccination coverage (28, 29). In addition, previous studies have also reported the positive impact of giving birth at home on incomplete immunization (23, 25, 38). This finding might be attributable to mothers gaining awareness of the health benefits of immunization through health education programs in the facility where they are going to give birth and through frequent counseling by healthcare professionals regarding having fully immunized children to protect children from VPDs, which is a common cause of death among children. In addition, mothers have a chance to vaccinate their children against polio and can have information on the subsequent schedule.

Women who had low media exposure had a significant positive association with unimmunized children. A number of studies have also reported the positive impact of media exposure on incomplete immunization (23, 25). The role of information in choosing the best strategical intervention to improve the immunization service was obvious, which is supported by different findings and governmental reports. When mothers understand the benefits of immunization, their interest in availing the service increases. Therefore, advertisements are circulated through mass media, which enables them to gain better knowledge about immunization.

Women whose relationship with the household head was that of daughters and others had a positive significant association with unimmunized children. A number of studies have also reported the positive impact of women having a relationship with the household head as daughters and others on vaccination coverage. If the relationship with the household head is not intimate, the communication between the head and the caregiver can be less interactive. In addition, women are too busy to handle plenty of activities, which leads them to forget immunization schedules. Furthermore, since they are less influential than head caregivers and are taking care of household activities as well as immunization schedules, there is a possibility that they are likely to miss the scheduled immunization day.

On the other hand, the community-level factors place of residence, distance from the health facility, media exposure, and community poverty level were significantly associated with unimmunized children. Mothers who perceived distance from the health facility as a big challenge were more likely to have unimmunized children than those who did not. Previous studies conducted in Ethiopia and in other countries have also reported the positive impact of being distant from health facilities on incomplete immunization (24, 26, 33, 39, 40). This finding might be attributable to the fact that when women are far away from the vaccination site, they may face transportation challenges, which may make them miss the scheduled vaccination date.

Mothers from rural residences were more likely to have unimmunized children than those from urban residences. Studies conducted in Ethiopia and in other countries have also reported the positive impact of being a rural resident on incomplete immunization and the negative impact on vaccination coverage (38). This finding is attributable to the fact that rural women may not have access to education regarding child health services. Furthermore, they may face challenges in accessing health facilities, which might lead to having unimmunized children.

Mothers from high-poverty communities were more likely to have unimmunized children than those from low-poverty communities. The intensity of poverty and the lifestyle of the community can affect the vaccination status of children. High-poverty communities with a low wealth index can result in unimmunized children, as people who are poor may prioritize daily activities, the food they are going to have, and other acute problems rather than unobservable health problems. In addition, people who are poor, on most occasions, tend to worry more about the daily need for food supply than about vaccination of children.

### Strengths and limitations of the study

The findings of this study can be generalized to all children aged less than 5 years in Ethiopia. These findings are dependent on the quality of the DHS program. The DHS collects a huge amount of information on households, important childhood health indicators, and maternal health in developing countries and is considered one of the reliable sources of information on maternal and child health. Thus, the findings of this study can be generalized for the source population of the participants and can be used as reliable evidence.

In this study, a multilevel analytical approach was used, which enabled the identification of a multilevel determinant of unimmunized children and provided insights for the Ethiopian Federal Ministry of Health to design appropriate interventions that reduce the proportion of unimmunized children in the country, which is a major indicator of a healthy childhood. Furthermore, the results of this study are representative of the Ethiopian population since appropriate estimation adjustments, such as weighting, were carried out before analysis.

This study has some limitations. First, the analysis was conducted using potential variables extracted from the DHS dataset. However, variables other than those mentioned in the DHS dataset could also be important factors associated with unimmunized children. Some of these variables may include distance to health service centers and quality of immunization services. Second, the cross-sectional survey data used in this study make it more difficult to conclude causality. Therefore, it is necessary to use longitudinal data that have been collected several times to confirm the validity of the observed associations.

## Conclusion

This study found a high prevalence of unimmunized children in Ethiopia, among whom nearly one-fourth of children did not get basic vaccinations.

Among individual factors, media exposure, place of delivery, and relationship with the household head were significantly associated with unimmunized children. Place of residence, distance from the health facility, community media exposure, and community poverty were the community-level factors significantly associated with unimmunized children.

In conclusion, immunization plays a vital role in improving public health in developing countries. It not only protects individuals from preventable diseases but also has significant societal and economic benefits. Implementation of effective immunization programs is crucial for reducing morbidity and mortality rates, promoting overall wellbeing, and advancing the development of a country.

Extending immunization programs can act as a catalyst for positive economic development and institutional changes at both the local and global levels. It is an investment for the well-being of communities, which contributes to a more prosperous and sustainable future.

## Data Availability

The raw data supporting the conclusions of this article will be made available by the authors, without undue reservation.

## Footnotes

^1^ http://dhsprogram.com
